# Optimized Adipogenic Differentiation and Delivery of Bovine Umbilical Cord Stem Cells for Cultivated Meat

**DOI:** 10.3390/gels10080488

**Published:** 2024-07-24

**Authors:** Derya Ozhava, Kathleen Lee, Cemile Bektas, Anisha Jackson, Krishi Patel, Yong Mao

**Affiliations:** Laboratory for Biomaterials Research, Department of Chemistry and Chemical Biology, Rutgers University, 145 Bevier Rd., Piscataway, NJ 08854, USA; do311@chem.rutgers.edu (D.O.); kwl49@scarletmail.rutgers.edu (K.L.); cemilekilic87@gmail.com (C.B.); atj43@scarletmail.rutgers.edu (A.J.); knp108@scarletmail.rutgers.edu (K.P.)

**Keywords:** adipogenic differentiation, bovine, umbilical cord stem cells, GelMA, GelMA microparticles, cultivated meat

## Abstract

Cultivated meat, also known as cell-based or clean meat, utilizes mesenchymal stem cells to cultivate mature cell types like adipocytes, which are pivotal for imparting the desired taste and texture. The delivery of differentiated cells, crucial in cultivated meat production, is facilitated through extensive exploration of 3D culturing techniques mimicking physiological environments. In this study, we investigated the adipogenic differentiation potential of bovine umbilical cord stem cells (BUSCs), sourced from discarded birth tissue, and assessed the feasibility of delivering differentiated cells for cultivated meat using gelatin methacrylate (GelMA) as a carrier for adipose tissue. Various adipogenic inducers, previously reported to be effective for human mesenchymal stem cells (hMSCs), were evaluated individually or in combination for their efficacy in promoting the adipogenesis of BUSCs. Surprisingly, while the traditional adipogenic inducers, including insulin, dexamethasone, isobutylmethylxantine (IBMX), indomethacin, and rosiglitazone, showed no significant effect on the adipogenic differentiation of BUSCs, efficient differentiation was achieved in the presence of a fatty acid cocktail. Furthermore, we explored methods for the delivery of BUSCs. Differentiated cells were delivered either encapsulated in GelMA hydrogel or populated on the surface of GelMA microparticles (MPs) as the adipose component of cultivated meat. Our findings reveal that after adipogenic induction, the lipid production per cell was comparable when cultured either within hydrogel or on MPs. However, GelMA-MPs supported better cell growth compared to hydrogel encapsulation. Consequently, the overall lipid production is higher when BUSCs are delivered via GelMA-MPs rather than encapsulation. This study not only systematically evaluated the impact of common adipogenic inducers on BUSCs, but also identified GelMA-MPs as a promising carrier for delivering bovine adipocytes for cultivated meat production.

## 1. Introduction

Cultivated meat, produced from animal cells through cell culture and tissue engineering technologies, accurately replicates the composition of real animal meat at a cellular level. Research has documented the utilization of embryonic, mesenchymal, and induced pluripotent stem cells as cell sources for the production of cultivated meat [1,2,3,4,5,6]. However, mesenchymal stem cells (MSCs) are often chosen as the cell source for their ability to self-renew, lineage-specific differentiation, and comparatively easy isolation [7]. To produce cultivated meat, cells are proliferated and then differentiated into specified mature cell types with specialized functions [6] such as myocytes (for muscle) and adipocytes (for fat) [8], in the presence of a scaffold [9]. With cell culturing and tissue engineering technologies, differentiated myocytes and adipocytes are assembled to resemble traditional meat, ultimately aiming to produce a meat product mimicking traditional meat without compromising the taste and texture experienced from traditional meat [10]. Although muscle fibers are the predominant part of meat products, lipids or fat make an important contribution to better flavor, texture, and nutrition [7,8,11,12].

Adipogenesis is a complex and multi-step process to form fat or adipose tissue, in which stem cells are differentiated into adipocyte precursor cells and further mature to become adipocytes [13]. MSCs with adipogenic potential are defined as preadipocytes and are a promising source of replicative cells for cultured fat. Existing protocols for adipogenesis of preadipocytes show variation in terms of both the differentiation cocktail and induction time. A detailed literature survey shows that most protocols use common inducers such as insulin, dexamethasone, isobutylmethylxantine (IBMX), indomethacin, and rosiglitazone, which have specific enhancing effects on adipogenesis [14,15,16]. For example, high concentrations of insulin are widely utilized to initiate the proliferation and differentiation of preadipocytes by mimicking insulin-like growth factor-1 in combination with dexamethasone and IBMX [14,17,18]. Dexamethasone, an anti-inflammatory steroid molecule, induces both osteocyte and adipocyte differentiation in a time- or dose-dependent manner [19,20]. The combination of IBMX with dexamethasone promotes adipogenesis by regulating peroxisome proliferator-activated receptor gamma (PPAR*γ*) [21]. However, due to safety concerns regarding some adipogenic substituents (such as IBMX), recent studies have explored the use of natural fatty acids that can activate PPAR*γ* at elevated concentrations for adipogenesis. For example, Mehta and co-workers’ study demonstrated the results of the adipocyte differentiation of bovine stromal vascular cells in the presence of a cocktail composed of seven different fatty acids [22].

While two-dimensional (2D) cell culture environments are the common practice of cell culture and differentiation processes, they do not adequately resemble the physiological environment of most cell types in tissues [23]. Therefore, three-dimensional (3D) culturing has been intensively explored to yield an environment more similar to the structural arrangement of cells in vivo [24,25,26,27]. Given that terminally differentiated adipocytes cannot be enzymatically detached and reseeded onto meat constructs, it is preferable for adipogenic differentiation to occur ‘in situ’. In this context, the 3D scaffolds that support the culturing and differentiation of adipogenic cells can function as carriers or delivery vehicles for the resultant differentiated adipocytes. In recent years, 3D cultivation techniques, including the applications of natural and synthetic scaffolds [28,29,30] (scaffold-free models and scaffold-based hybrid model systems), have been used in cell studies [31,32]. Natural polymers such as gelatin have been widely investigated to provide a 3D environment for cell culturing [33]. Gelatin, the denatured form of collagen, has low immunogenicity and high biocompatibility [34] and can be readily modified by methacrylation and photo crosslinked under UV light [35]. Gelatin methacrylate (GelMA) is a photo-crosslinkable derivative of gelatin, characterized by the incorporation of methacrylate groups onto the gelatin backbone. This modification results in GelMA exhibiting biocompatible properties akin to those of gelatin, facilitated by their similar molecular structures [36]. Notably, GelMA, featuring natural cell-binding motifs analogous to those found in gelatin, facilitates enhanced cell adhesion and secretion of matrix metalloproteinases that are crucial factors in extracellular matrix (ECM) remodeling. GelMA can be directly used in the form of hydrogel or converted to hydrogel microspheres; both provide a 3D culture environment for cells [37]. While cell entrapment in GelMA hydrogel has traditionally been a common cell delivery method [38], we hypothesize that the increased surface areas and easy nutrient exchange offered by GelMA microparticles (GelMA-MPs) make them a better candidate as cell carriers for adipocytes.

The initial and crucial phase in cultured meat research is the selection of an appropriate starting cell type, a decision that significantly impacts subsequent processes [39]. Although embryonic, induced pluripotent, and immortalized cell lines exhibit the advantage of unlimited proliferative capacity, they pose challenges related to regulatory compliance, consumer acceptance, and ethical considerations [40]. The ideal cells should be readily and efficiently isolable from the source, exhibit robust proliferation in cost-effective media, and be amenable to straightforward differentiation protocols [41]. For the production of cultured fat, MSCs represent a viable option. MSCs have been effectively isolated from bone marrow, adipose tissue, and placental tissues [7]. Even though biopsies constitute minor injuries to animals, to prevent any harm, alternative cell sources should be explored. Stem cells isolated from birth tissues, such as the placenta, which are frequently discarded as medical waste, represent a potentially sustainable source of cells. Bovine placental tissue, specifically umbilical cords, provides a sustainable and non-invasive source for cell isolation. MSCs isolated from bovine umbilical cords (BUSCs) expressed MSC cell surface markers and showed multilineage differentiation potentials, including adipogenic differentiation [42]. This study aims to determine the optimal adipogenic differentiation formulation for BUSCs and explore their potential delivery when differentiated in/on GelMA for cultivated meat. Initially, various adipogenic differentiation formulations were prepared, including commercial formulations and compositions consisting of typical adipogenic inducers—dexamethasone, IBMX, insulin, indomethacin, rosiglitazone, and fatty acids—commonly used for human bone marrow mesenchymal stem cells (hMSCs). These formulations were tested on BUSCs to assess their effectiveness on adipogenic differentiation. The systematic testing revealed that the natural fatty acids, comprising seven distinct types of acid, exhibited a more robust impact on the adipogenic differentiation of BUSCs. Interestingly, the commercial formulations optimized for hMSCs promoted the adipogenic differentiation of hMSCs but had a minimal effect on the adipogenic differentiation of BUSCs. The adipogenic inducers, whether tested individually or in combination, demonstrated limited efficacy in enhancing the adipogenic differentiation of BUSCs. Furthermore, considering the critical role and benefits of 3D scaffolds in promoting the culture and differentiation of adipogenic cells, alongside their capacity to carry and deliver differentiated adipocytes, we have investigated the delivery of BUSCs encapsulated within GelMA hydrogel and GelMA-MPs. The growth and adipogenic differentiation of BUSCs on these 3D culturing systems were monitored and compared. While the lipid production of stem cells remained comparable when cultured in GelMA hydrogel or on the surface of GelMA-MPs, cells on GelMA-MPs exhibited more robust growth and yielded a higher quantity of lipids during the same culturing period. This study represents the first systematic comparison of various adipogenic inducers and identified a potential carrier for delivering bovine adipocytes.

## 2. Results and Discussion

**Figure 1:**
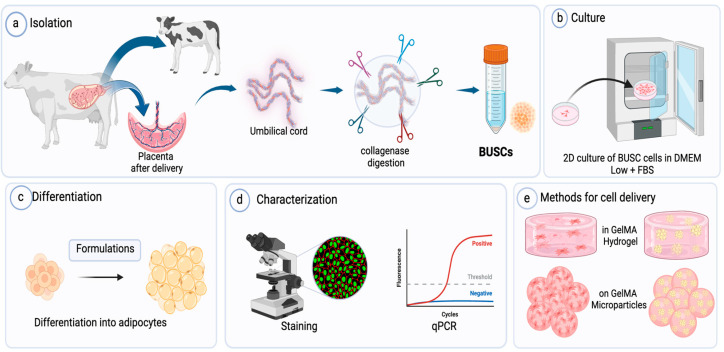
Study design. The major steps involved in culturing bovine adipocytes for cultivated meat. (**a**) Isolation of BUSCs from bovine umbilical cord tissues, (**b**) culturing of isolated cells in an incubator at 37 °C with 5% CO_2_ and 95% humidity, (**c**) optimization of adipogenic differentiation media for BUSCs, (**d**) characterization of adipogenic differentiation by staining and qPCR analysis, (**e**) identification of a delivery method for bovine adipocytes. BUSCs: bovine umbilical cord stem cells, GelMA: gelatin methacrylate.

Stem cells derived from bovine umbilical cords (BUSCs) have shown multilineage differentiation capabilities [43]. This study introduces the utilization of these cells obtained from discarded birth tissue, without harming animals, as a sustainable cell source for making cultivated meat. Figure 1 is a schematic representation of the study design with the ultimate goal of achieving highly effective adipogenic differentiation and optimal delivery of adipocytes to produce a cultivated meat construct. Our methodology comprised the following stages: (a) isolation of BUSCs from discarded birth tissue, (b) cultivation of these cells on tissue-cultured cell plates (TCPs), (c) refinement and optimization of adipogenic induction media tailored for BUSCs to facilitate the generation of adipose tissue, (d) characterization of adipogenic differentiation with multiple methods, and finally, (e) identification of an effective delivery method for differentiated bovine adipocytes.

### 2.1. Determination of an Adipogenic Differentiation Cocktail for BUSCs

Bovine stromal cells isolated from the umbilical cord (BUSCs) were extracted from Angus cow placental tissues following the methodology outlined in our prior publication [40]. These cells exhibited multilineage differentiation potentials and retained their capabilities for expansion over several passages without compromising their differentiation capacities.

**Figure 2:**
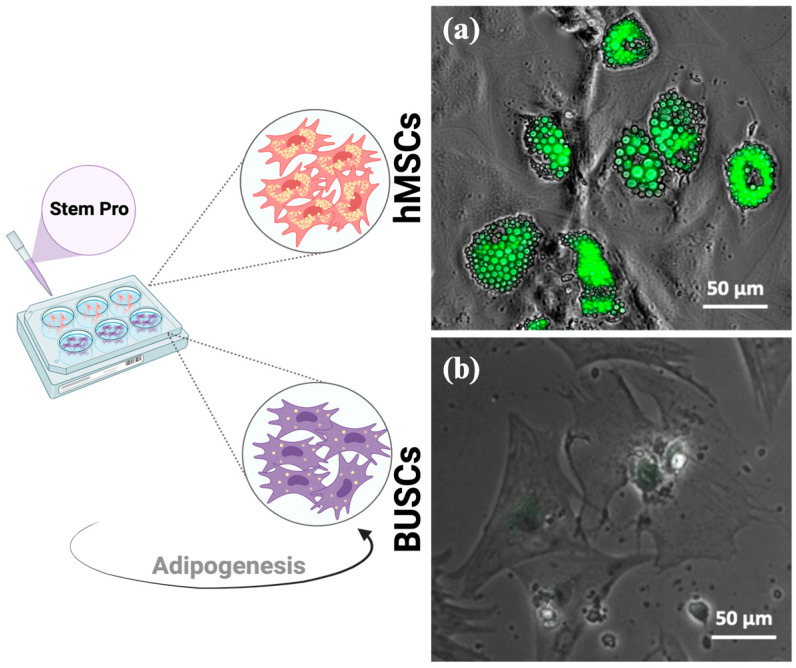
Adipogenic induction of hMSCs and BUSCs with StemPro adipogenic differentiation kit. (**a**) hMSCs or (**b**) BUSCs were induced with StemPro medium for 20 days, followed by LipidTOX staining. Green fluorescent and phase contrast images were captured using ECHO fluorescent microscope. Representative merged images are shown. Scale bar = 50 µm.

To induce the adipogenic differentiation of BUSCs, StemPro adipogenesis differentiation kit medium, typically optimized for human mesenchymal stem cells (hMSCs), was initially tested. Both BUSCs and hMSCs were induced with StemPro adipogenic differentiation media for 20 days. To assess the lipid droplet formation, cells were fixed and stained with LipidTOX, which specifically stains neutral lipids. Following induction, numerous LipidTOX stained lipid droplets were observed in hMSCs (Figure 2a). Interestingly, minimal LipidTOX positive staining was evident in BUSCs treated with StemPro medium (Figure 2b). While StemPro adipogenic medium has been utilized to prompt differentiation in bovine stem cells previously, this study marks the first direct comparison of its efficacy between human and bovine stem cells. Although StemPro is optimized for hMSCs, the formulation’s efficiency on BUSCs suggests a need for further exploration and optimization of adipogenic media for this specific cell type.

Adipogenic differentiation has been extensively studied in vitro using murine stem cells and human stem cells [22]. The adipogenic process was determined to be initiated by the activation of transcription factors, namely peroxisome proliferator-activated receptor gamma (PPAR*γ*) and CCAAT/enhancer-binding protein alpha (C/EBP-*α*) [22,44]. Various compounds have been tested for their inductive effect on adipogenic differentiation. Among them, dexamethasone, indomethacin, IBMX, insulin, and rosiglitazone have been identified as adipogenic inducers [22,45,46]. The majority of studies reported in the literature used a complete medium comprising DMEM supplemented with 10% FBS and additional components such as dexamethasone, indomethacin, IBMX, insulin, or rosiglitazone. Based on the information we gathered from the literature, nine differentiation formulations were designed to identify key inducers of adipogenic differentiation for BUSCs. The composition of these nine formulations has been listed in Table 1.

**Figure 3:**
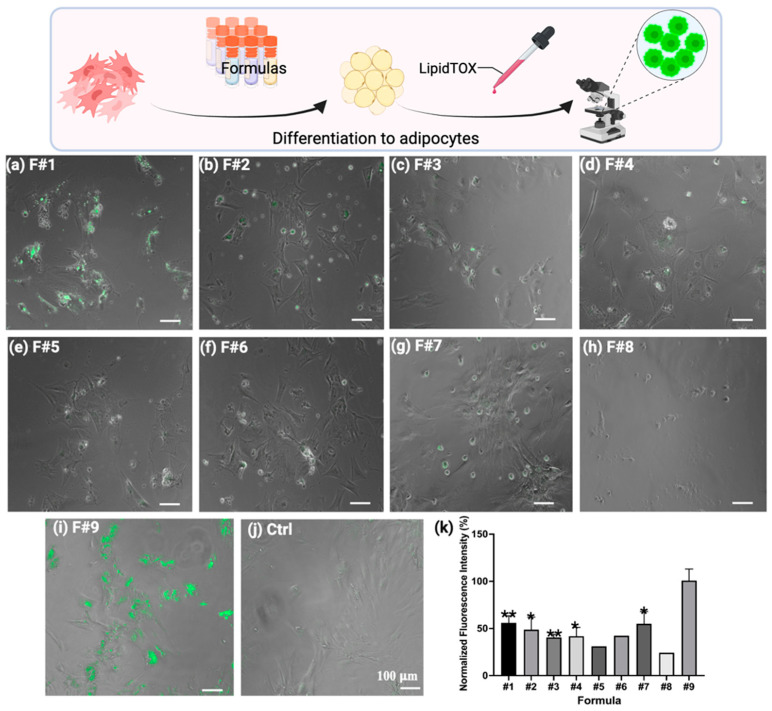
Adipogenic differentiation of BUSCs induced by 9 different induction formulations. BUSCs were induced for 24 days with Formulas #1 to #8 (**a**–**h**) and 7 days with F#9 (**i**) and stained with LipidTOX. Representative merged images of fluorescent images and phase contrast images are shown (**a**–**j**). (**k**) The fluorescent intensities of multiple images from each condition were measured using ImageJ software and subsequently normalized to control (no induction). Data shown are mean ± SD. Statistical analysis was performed for each group in comparison with F#9. * *p* < 0.05 and ** *p* < 0.01 (n ≥ 3 except for F#5 and F#8, where limited cells were captured). Scale bar = 100 μm.

Adipogenic differentiation of BUSCs was initiated using various differentiation media over a period of 24 days. Upon completion, the efficacy of adipogenic differentiation was assessed by observing the formation of lipid droplets within cells. Cells were fixed and stained with LipidTOX dye, which selectively stains neutral lipids [11,47]. Notably, BUSCs cultured in Formula #1 showed noticeable accumulations of lipid droplets (Figure 3a), whereas cells differentiated in Formulas #2 to #7 did not exhibit significant lipid droplet formation (Figure 3b–g). Consistent with our previous observation, Formulas #5 and #8 (both containing StemPro adipogenic kit) were shown to be ineffective in inducing lipid accumulation. Additionally, the viability of BUSCs, as indicated by the number of adhered cells, decreased with prolonged exposure to StemPro (Figure 3e), and this decline worsened in the presence of additional inducers (Figure 3h). On the other hand, BUSCs induced by Formula #9 (F#9) displayed prominent lipid formation on day 7 (Figure 3i). The visual observation was further corroborated by quantifying the total fluorescent intensities of multiple images for each condition (Figure 3k). The formation of oil droplets in the presence of F#9 on day 7 was significantly higher compared to that induced by other formulas.

**Figure 4:**
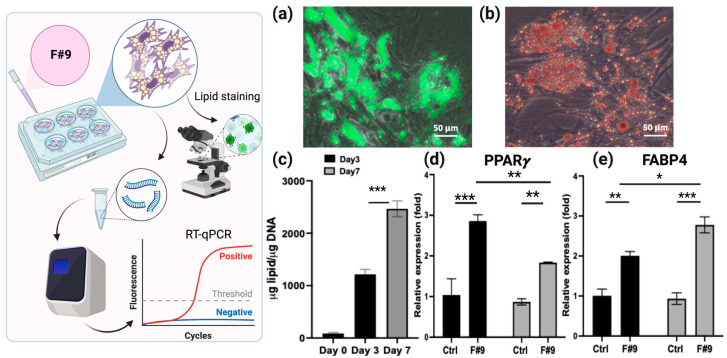
Characterization of adipogenic differentiation of BUSCs induced by F#9. BUSCs were induced by F#9 for 7 days. (**a**) Representative merged image of LipidTOX staining and its corresponding phase contrast. (**b**) Representative merged image of Oil Red O staining and its corresponding phase contrast image. (**c**) Quantification of neutral lipid in cells induced for 3 and 7 days. The relative expression of PPAR*γ* gene (**d**) and FABP4 gene (**e**) in BUSCs treated with F#9 or growth medium (uninduced control) for 2 and 7 days. Data shown are mean ± SD (n = 4) * *p* < 0.05, ** *p* < 0.01, *** *p* < 0.005. Scale bar = 50 µm.

F#9, consisting of a combination of seven fatty acids, has been used to induce the adipogenic differentiation of bovine adipose-derived stem cells alongside adipogenic inducers [22]. Remarkably, in this investigation, F#9 promoted rapid and efficient adipogenic differentiation even in the absence of other inducers. Oil droplet formation induced by F#9 was readily observable as early as day 3, with cells demonstrating sustained growth and differentiation in its presence. BUSCs induced by F#9 for 7 days exhibited strong green fluorescent LipidTOX staining (Figure 4a). To validate the lipid staining, a common lipid staining technique using Oil Red O was also performed on cells induced by F#9 (Figure 4b). Both LipidTOX staining and Oil Red O staining confirmed the accumulation of lipid droplets in induced BUSCs. Additionally, neutral lipid production was extracted and quantified using a fluorometric assay (Figure 4c). The progressive increase in lipid accumulation over time corroborated the findings observed via LipidTOX staining and fluorescent intensity quantification.

To validate the activation of adipogenic pathways, the expression of adipogenic markers [48] in BUSCs was assessed via qPCR analysis after induction for 2 and 7 days (Figure 4d,e). Specifically, the expressions of two markers were analyzed: PPAR*γ* (peroxisome proliferator-activated receptor gamma), an early marker of adipogenic differentiation, and FABP4 (fatty acid binding protein 4), a later marker involved in the maturation of adipocytes [49]. PPAR*γ* serves as the initiator of adipogenic differentiation, regulating downstream gene expression crucial for adipogenic differentiation. Typically, its expression rises at the onset of adipogenic differentiation and declines as adipocytes mature [49,50]. The expression of PPAR*γ* in BUSCs treated with F#9 increased on day 2 and decreased to a level comparable to the control (uninduced cells) on day 7 (Figure 4d). The stimulated expression of FABP4 in the presence of F#9 was only observed on day 7 (Figure 4e). The declined expression of PPAR*γ* and increased expression of FABP4 suggest that by day 7, adipogenic differentiation had likely progressed into the maturation phase of adipocytes.

### 2.2. Determination of Optimized F#9 Concentration for BUSC Adipogenesis

**Figure 5:**
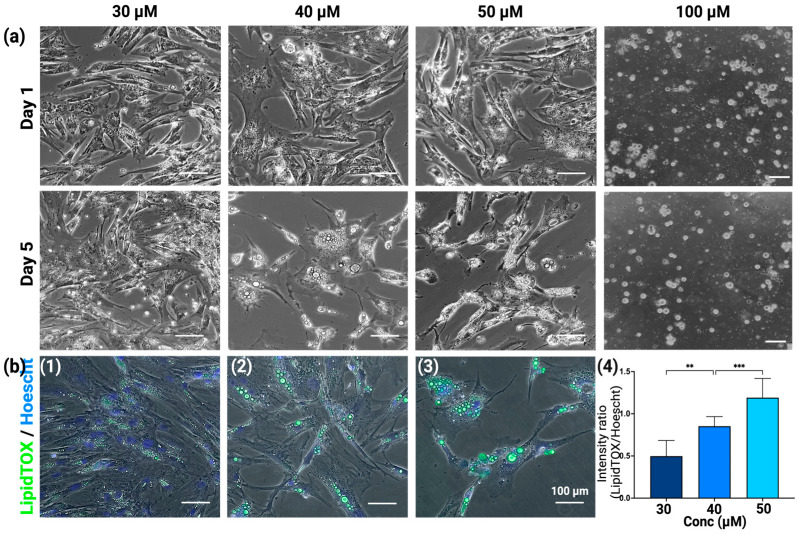
Adipogenic induction of BUSCs with F#9 at different concentrations. (**a**) The lipid droplets were visualized with phase imaging on day 1 and day 5. (**b**) The lipid droplets and nuclei were stained with LipidTOX (oil droplets, green) and Hoechst dye (nuclei, blue) after 7 days of induction. Scale bar = 100 μm. The green fluorescent intensity relative to the blue fluorescent intensity was measured using ImageJ software. Data shown are mean ± SD (n = 6) ** *p* < 0.01, *** *p* < 0.005.

To determine the optimal concentration of F#9 for the adipogenic differentiation of BUSCs, concentrations ranging from 10 μM to 200 μM were tested. Among these, 50 μM of F#9 (each component at 50 μM) exhibited the highest inductive activity, surpassing that of 30 μM and 40 μM (Figure 5a). Treatment with F#9 at a concentration higher than 50 μM (such as 100 μM) did not induce more oil droplet formation; instead, it led to cell death (Figure 5a). In pursuit of cost-effectiveness, lower concentrations of F#9 that induce the desired adipogenic differentiation are preferred. Therefore, F#9 concentrations at 30, 40, and 50 μM were further compared (Figure 5). As depicted in Figure 5a, BUSCs differentiated in the presence of F#9 at 30, 40, and 50 μM. However, the presence of oil droplets was more prominent, and they appeared to be larger, in cells treated with 50 μM. Cells were stained with LipidTOX for lipid content and Hoechst dye for DNA (indicating the number of cells) (Figure 5b(1)–(3)). The green fluorescent intensity relative to blue fluorescent intensity was measured using NIH Image J (Fiji for Mac OS X) (Figure 5b(4)). Consistent with observations, the lipid production per cell was highest in BUSCs treated with 50 μM F#9. Hence, the concentration of F#9 at 50 μM was selected as the optimal concentration for further studies on BUSCs.

### 2.3. Evaluation of Pretreatment Effects of Different Adipogenic Inducers on the Adipogenic Differentiation of BUSCs Induced by F#9

**Figure 6:**
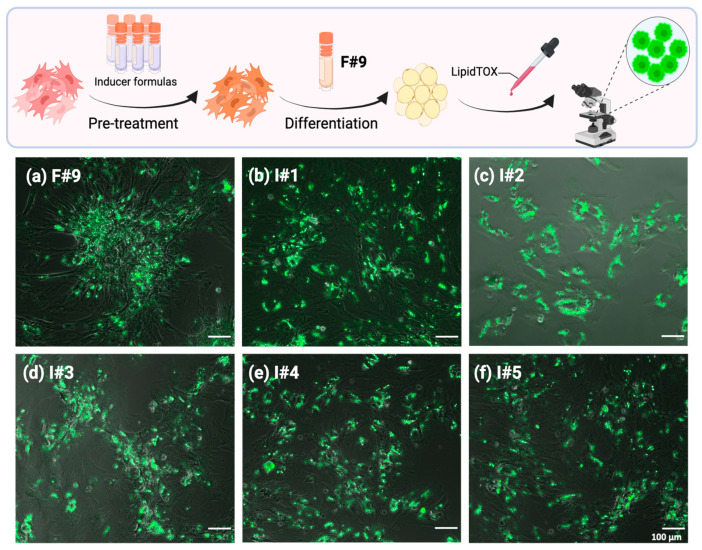
The effects of adipogenic inducers on the adipogenic differentiation of BUSCs induced by F#9. BUSCs pretreated with growth medium (control, (**a**)) or inducers: Formulas #1–#5 (**b**–**f**) for 8 days, followed by induction with F#9 for 5 days. Cells were fixed and stained with LipidTOX. Representative merged images are shown. Scale bar = 100 μm.

F#9 contains a mixture of fatty acids that rapidly and effectively promote the adipogenic differentiation of BUSCs (Figure 3, Figure 4 and Figure 5). The precise mechanism underlying this induction remains unclear, but it may be attributed to the seven fatty acids in this formulation, which serve as building blocks for lipid droplets. Additionally, certain fatty acids, such as oleic acid, have been shown to stimulate the PPAR*γ* pathway [51]. While other adipogenic inducers such as IBMX, insulin, indomethacin, and dexamethasone may induce adipogenic differentiation via additional pathways, it remains to be explored whether these inducers enhance the adipogenic differentiation of BUSCs induced by F#9. To assess the effects of inducers (IBMX, dexamethasone, indomethacin, or insulin), various combinations were prepared as detailed in Table 2. BUSCs were pretreated with these inducers for 8 days, followed by F#9 treatment for 5 days. Lipid droplets formed under each condition were stained with LipidTOX and imaged (Figure 6). Overall, there was no significant difference in green fluorescent intensities observed among different inducer pretreatments. Additionally, no enhanced formation of oil droplets was noted in pretreated groups compared to those not pretreated (F#9 induction only, see Figure 6a). It appears that the adipogenic differentiation induced by F#9 in BUSCs may have overshadowed the effects of the other inducers. Species differences may also contribute to the differential responses to inducers, as these inducers were identified using human cell models. The underlying mechanism behind the lack of adipogenic stimulation of BUSCs by common adipogenic inducers requires further investigation. Considering that some adipogenic inducers can be costly and may raise food safety concerns, inducing BUSCs in the absence of these inducers without compromising the yield of adipocytes would likely be a more favorable pathway for advancing cultivated meat production.

### 2.4. Delivery of BUSCs on GelMA Microspheres or in GelMA Hydrogels

Meat, though mainly consisting of muscle fibers, contains approximately 30% fat, which improves its flavor, texture, and nutritional profile [8,11]. Lipids and adipocytes are distinct components of food, with adipocytes storing a significant portion of lipids, contributing to the natural fat content of meat [52]. The co-culturing and co-differentiation of adipocytes and myocytes for cultivated meat production have yet to be achieved [53]. Presently, these two primary components of cultivated meat are grown independently and subsequently assembled for a short period of co-culturing [53]. For this purpose, BUSCs require carriers on which to grow, differentiate, and ultimately be integrated into the final meat construct.

**Figure 7:**
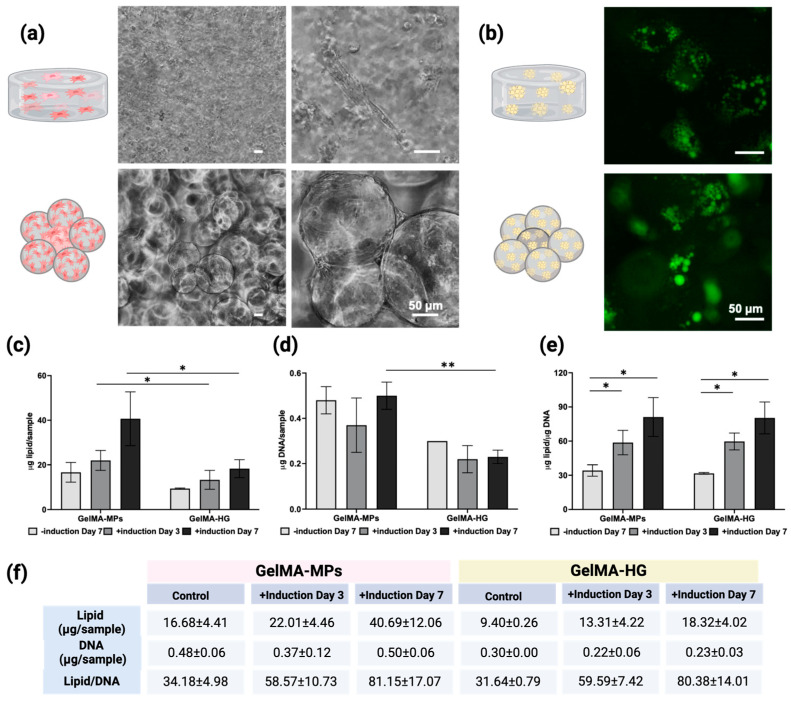
Culturing and adipogenic differentiation of BUSCs within GelMA hydrogels (GelMA-HG) and on GelMA-MPs with F#9. (**a**) Representative phase contrast images of BUSCs on day 4. (**b**) After induction for 7 days, cells in GelMA-HG and on GelMA-MPs were stained with LipidTOX (oil droplets, green). (**c**) Lipids were extracted, and the neutral lipid content was quantified. (**d**) The total DNA contents were quantified. (**e**) The relative lipid production normalized to DNA. (**f**) Table summarizes the results of the tests. Data shown are mean ± SD (n = 4) * *p* < 0.05, ** *p* < 0.01. Scale bar = 50 µm.

Methacrylated gelatin (GelMA) is a biocompatible material and has been used for culturing and delivering cells [54,55]. GelMA is synthesized through the reaction of methacrylic anhydride with the primary amine groups of gelatin at elevated temperatures [37]. This process results in the addition of methacrylate groups onto the gelatin backbone. The structural modifications in gelatin were assessed using ^1^H NMR [56,57], and the degree of substitution was quantified as 92 ± 1.4% through a ninhydrin assay, as detailed in our previously published methods [58]. Gelatin could be engineered into a 3D structure as a hydrogel (GelMA-HG) or microspheres (GelMA-MPs). Subsequently, cells seeded within GelMA-HG or on GelMA-MPs were cultured at 37 °C with 5% CO_2_ and 95% humidity. Figure 7a depicts the representative phase contrast images of cells in GelMA-HG and on GelMA-MPs, revealing robust cell growth with both GelMA constructs. To evaluate the adipogenic differentiation of cells in GelMA-HG or on GelMA-MPs, after culturing for 14 days, cells were induced with 50 μM F#9.

After induction for 7 days, the lipid accumulation in BUSCs either in GelMA-HG or on GelMA-MPs was visualized with LipidTOX staining (Figure 7b). To assess the lipid content retained in cells cultivated either in GelMA-HG or on GelMA-MPs, we conducted a fluorometric neutral lipid assay (Figure 7c). This method is preferred for lipid quantification due to its rapid response time and straightforward sample handling, coupled with high detection sensitivity and specificity for neutral lipids [59,60,61]. Meanwhile, the DNA contents of another set of samples were measured to normalize the lipid production per cell. While both GelMA-HG and GelMA-MPs exhibited a trend of time-dependent increase in lipid production, the total lipid contents of cells on GelMA-MPs were significantly higher than those in GelMA-HG on day 7 (Figure 7c). Total DNA quantification indicated a higher cell count on GelMA-MPs compared to that in GelMA-HG (Figure 7d). The observed higher cell growth on the surface of GelMA-MPs than within GelMA-HG may be attributed to easier nutrient access and waste exchange when cells are situated on the surface of MPs. However, when lipid production was normalized to DNA contents (Figure 7e), no significant difference was detected among different groups, suggesting that the adipogenic differentiation ability of BUSCs remains unaltered whether grown on the surface of GelMA-MPs or in GelMA-HG.

In addition to promoting better cell growth, GelMA-MPs exhibit greater moldability compared to GelMA-HG, which typically sets in a pre-defined mold. This moldability of GelMA-MPs offers versatility in the assembly and integration of fat tissue with muscle tissue for cultivated meat production.

## 3. Conclusions

In conclusion, this study highlights the potential and efficacy of utilizing BUSCs, sourced from typically discarded birth tissue, in adipose tissue formation for cultivated meat. To optimize the adipogenic differentiation of BUSCs, various adipogenic inducers identified in the literature were tested in different combinations. While a rapid and efficient adipogenic differentiation of BUSCs was achieved by using a fatty acid cocktail, the treatments of inducers did not significantly enhance the adipogenesis of BUSCs. Furthermore, GelMA facilitates the viability of BUSCs in both GelMA-HG and GelMA-MP forms. Notably, the proliferation of BUSCs is significantly enhanced on GelMA-MPs compared to in GelMA-HG, resulting in an increased cell population and, consequently, greater lipid accumulation. Therefore, GelMA-MPs, with their excellent moldability and compatibility with BUSCs, have been identified as a highly effective potential cell carrier for delivering the adipogenic component in cultivated meat production. In short, the utilization of a fatty acid cocktail streamlines the formulation of the adipogenic medium, reducing the need for various chemical inducers, which introduce complexity and safety concerns. From an economic perspective, employing typically discarded birth tissue, BUSCs, as a cell source, along with highly biocompatible and cost-effective 3D cell scaffolding, GelMA, potentially enhances the system’s viability for large-scale cultivated meat production.

## 4. Materials and Methods

### 4.1. Isolation of Bovine Umbilical Cord-Derived Stem Cells

The placental tissues from Angus cows were provided by the New Jersey Agricultural Experiment Station (Rutgers University). Bovine umbilical cord-derived stem cells (BUSCs) were extracted from cow placental tissues using established methods [42,62,63]. The detailed protocol for isolating BUSCs utilized in this study has been previously reported [43]. Briefly, after collagenase digestion, undigested tissue pieces were washed with PBS and then cultured in 10 cm cell culture dishes (Cat#229621, CELLTREAT, Pepperell, MA, USA) in DMEM (low glucose, Gibco, Waltham, MA, USA) complete medium (DMEM with 10% fetal bovine serum (FBS) + 1× antibiotic–antimycotic (Thermo Fisher, Branchburg, NJ, USA) for 7–10 days. The harvested cells were either sub-cultured or cryopreserved as BUSC P1 cells. Human mesenchymal stem cells (hMSCs) from bone marrow (passages 3–5, Texas A&M, 8011L, College Station, TX, USA) were cultured in alpha-MEM complete medium (alpha-MEM complete with 10% FBS + 1× antibiotic–antimycotic) for a duration of 7–10 days.

### 4.2. The Effect of a Commercial Adipogenesis Differentiation Kit on the Adipogenesis of BUSCs

The commercial adipogenesis differentiation kit developed for human mesenchymal stem cells, StemPro (A1007001, Gibco, Waltham, MA, USA), was initially tested for its effectiveness on BUSC adipogenesis. BUSCs (passages 2–4) were grown to a confluency of 80%, trypsinized using trypsin–EDTA (0.25%, Gibco, Waltham, MA, USA), and then counted with a hemocytometer. In a 24-well plate, cells were seeded at a density of 2 × 10^4^ cells per well. hMSCs were used as a control and seeded at 2 × 10^4^ cells/well. Following overnight incubation in a cell culture incubator set at 37 °C with 5% CO_2_ and 95% humidity, StemPro was added to each well. Following the induction for 21 days, cells were fixed with a 4% paraformaldehyde (PFA) solution in PBS (Thermo Fisher Scientific, Waltham, MA, USA) and washed with phosphate-buffered saline (PBS, Cytiva, Marlborough, MA, USA). A stock solution of LipidTOX (LipidTOX, InvitrogenTM, Waltham, MA, USA) was prepared by diluting the LipidTOX neutral lipid stains at a ratio of 1:400 in PBS. For intracellular triglyceride lipid droplet staining, a 300 μL working solution of LipidTOX was added to each well and incubated at room temperature for at least 30 min. Following staining, the wells were rinsed twice with 300 μL of PBS. The stained lipid droplets were fluorescently visualized using an ECHO microscope (Echo Revolve, San Diego, CA, USA).

### 4.3. Different Cocktails for Adipogenic Differentiation of BUSCs

To test various adipogenic formulations, nine different formulas were prepared (Table 1). Formulas #1–#8 are various combinations of inducers that are commonly used in the literature for the adipogenesis of MSCs, such as isobutylmethylxantine, dexamethasone, insulin (Sigma-Aldrich, Burlington, MA, USA), indomethacin, and rosiglitazone (VWR, Radnor, PA, USA). Formula #9 is composed of the combination of seven fatty acids from Cayman Chemical: myristoleic acid (Cayman Chemical, Ann Arbor, MI, USA), phytanic acid, elaidic acid, oleic acid, palmitoleic acid, erucic acid (Sigma-Aldrich, Burlington, MA, USA), and pristanic acid (Toronto Research Chemicals, North York, Canada). In a 24-well plate, BUSCs were seeded at a density of 2 × 10^4^ cells per well and incubated in a cell culture incubator. After a 24 h incubation period, adipogenic induction media, each formulated with one of the nine distinct adipogenic formulas, were administered to the designated wells. The media were changed every 3 days over a 24-day period. After the completion of adipogenic differentiation, the cells were fixed with a 4% PFA solution, washed two times with PBS, and stained with LipidTOX. Fluorescent images were captured using an ECHO fluorescent microscope (Echo Revolve, San Diego, CA, USA). The settings for LipidTOX (FITC channel) were an exposure of 100 ms, low gain, and 100% light intensity. The settings for DNA staining (DAPI channel) were an exposure of 125 ms, low gain, and 100% light intensity. Each image’s TIFF file was exported and opened in FIJI software (NIH ImageJ2 version 2.14.0/1.54f). The total fluorescent intensity of each image was measured using ImageJ.

### 4.4. Quantitative PCR (qPCR) Analyses

The quantification of the relative expression levels of the transcripts encoding the adipocyte-specific gene markers (PPAR*γ* and FABP4) was assessed by performing qPCR analysis. Briefly, cDNA synthesis was conducted according to the manufacturer’s protocol (Promega, Madison, WI, USA), after the purification of RNA from lysates using the SV 96 Total RNA Isolation System (Promega, Madison, WI, USA) and concentration determination of RNA using a TECAN Spark Nano plate (TECAN, Männedorf, Switzerland). qPCR analysis was performed using the Light Cycler 480^®^ system (Roche, Basel, Switzerland) following a standard procedure. The qPCR primer sets for cow GAPDH (reference gene), PPAR*γ*, and FABP4 were purchased from Qiagen (Germantown, MD). Each testing condition included 3–4 biological replicate samples, and every sample was subjected to duplicate runs. Following the completion of the runs, a second derivative analysis was conducted on the raw data to ascertain the mean Cp (crossing point PCR cycle) for each sample. The mRNA expression in relation to control conditions was determined through Pfaffl analysis:

Relative gene expression = 2^(∆Cp target)/2^(∆Cp reference)(1)

### 4.5. Determination of Optimal F#9 Concentration for the Adipogenesis of BUSCs

BUSCs were seeded at 2 × 10^4^ cells per well in 24-well plates. Following overnight incubation, the cells were induced with 30, 40, 50, or 100 μM of F#9, composed of seven different fatty acids, in complete media for 7 days. After the 7-day induction period, cells were fixed in the presence of 4% PFA and subsequently washed with PBS twice. Fixed cells were then stained with LipidTOX at 1:400 dilution and Hoechst dye 33258 (Cat #83219, AnaSpec Inc., Fremont, CA, USA) at 1:500 dilution for 30 min. After staining, cells were washed twice with PBS and examined using the ECHO fluorescent microscope. The fluorescent images (TIFF) were captured and the fluorescent intensities of LipidTOX and Hoechst dye were measured using FIJI software (NIH).

### 4.6. Pretreatment Effect of Different Adipogenic Inducers on the Adipogenic Differentiation of BUSCs in F#9

BUSCs were seeded at 2 × 10^4^ cells per well in 24-well plates. After overnight incubation, cells were induced with different formulations (Table 2) with different combinations of adipogenic inducers for 8 days. After pretreatment, cells were induced with F#9 for 5 days. The medium was changed every three days. Following the induction with F#9, cells were fixed with 4% paraformaldehyde, stained with LipidTOX, and examined using the fluorescence microscope.

### 4.7. Procedure for Gelatin Methacrylation

The methacrylation of gelatin was conducted following the published synthesis method [57], which involves the preparation of a 10% (*w*/*v*) gelatin solution (type A from bovine skin, 300 Bloom, Sigma-Aldrich, MO, USA) in 100 mL of 0.25 M, pH 9.4 carbonate–bicarbonate buffer (Sigma-Aldrich, Burlington, MA, USA). After dissolving gelatin at 55 °C, 0.938 mL of methacrylic anhydride (Sigma-Aldrich, Burlington, MA, USA) was introduced into the solution under constant magnetic stirring at 500 rpm to achieve a targeted 100% degree of substitution. The reaction procedure was carried out for 1 h at 55 °C and then terminated by lowering the pH of the solution to 7.4. The resulting solution containing methacrylated gelatin (GelMA) was filtered, dialyzed for 2 days, and subsequently lyophilized. The white solid product obtained was stored at 4 °C until use. The degree of substitution was determined by examining the ^1^H-NMR spectra of both gelatin and GelMA, and performing a ninhydrin assay as described in our previous study [58].

### 4.8. Preparation of GelMA Hydrogel Molds and Seeding of BUSCs in GelMA Hydrogels

Cell-laden GelMA hydrogels (GelMA-HG) were prepared on polydimethylsiloxane (PDMS) (Slygard 184, Dow Corning, Midland, MI, USA) molds as described before [35]. The PDMS templates, which contain the negatives of discs (8 mm in diameter) were sterilized under UV light for 30 min.

A 10% GelMA solution (*w*/*v*, in complete medium) was prepared in the presence of 0.05% LAP photo initiator (lithium phenyl-2,4,6-trimethylbenzoylphosphinate) (Sigma-Aldrich, Burlington, MA, USA) (*w*/*v*). BUSCs were collected and centrifuged, and the pellet was resuspended in the GelMA solution at a concentration of 2 × 10^6^ cells/mL. Thirty μL of cell-laden solution was poured into the PDMS molds and crosslinked for 40 s using a 405 nm light source (Sovol, Shenzhen, China) at 20 mW cm^−2^ and at a distance of 13 cm. The crosslinked hydrogels were transferred to a 48-well cell-repellent plate (Greiner Bio-One, Kremsmünster, Austria) filled with growth medium, followed by two medium washes, and then incubated in the same medium. Growth medium was changed every 3 days throughout 14 days.

### 4.9. Synthesis of GelMA Hydrogel Microparticles (GelMA-MPs) and Seeding of BUSCs

The GelMA hydrogel microspheres (GelMA-MPs) were prepared by following the published synthesis method [54]. Briefly, the 10% (*w*/*v*) GelMA solution was prepared in 5 mL water at 37 °C in a falcon tube. Next, 1.0 mL of Tween-20 (Thermo Fisher Scientific, Waltham, MA, USA) was added into 75 mL of mineral oil (Sigma-Adrich, St. Louis, MO, USA) preheated to 37 °C in a three-neck round-bottomed flask (250 mL). Then, the GelMA solution mixed with 14 mg of ammonium persulfate (Sigma-Aldrich, Burlingtin, MA, USA) was dripped into the mineral oil phase. The obtained water–oil emulsions were further mixed at 400 rpm for 5 min; then, the system was deoxygenated by bubbling N_2_ gas through the emulsion for 20 min. The temperature of the system was elevated to 100 °C under constant stirring and kept at that temperature for 40 min to ensure thermal crosslinking of microspheres. The particles obtained at the end were separated from the reaction solution by centrifugation and washing with distilled water. The particles were frozen at −20 °C, lyophilized, and stored at 4 °C until further use.

Three mg/well of lyophilized GelMA-MPs were individually weighed into a 48-well cell-repellent plate and sterilized under UV for 30 min. The GelMA-MPs were resuspended in 200 μL of DMEM complete medium and rotated at 150 rpm for 2 h to rehydrate. BUSCs were harvested and resuspended in culture medium at 30 × 10^4^ cells/mL, and 200 μL of cell solution was pipetted into each well containing 3 mg of GelMA-MPs for a final cell concentration of 6 × 10^4^ cells/well. The plates were incubated at 37 °C with 5% CO_2_ and 95% humidity for 14 days, with medium changes every 3 days, and shaking at 200 rpm starting on day 2 (Benchmark Scientific, Sayreville, NJ, USA).

### 4.10. Adipogenic Differentiation of BUSCs on GelMA-MPs or in GelMA-HG

After culturing BUSCs on GelMA-MPs or in GelMA-HG for 14 days, the process of inducing adipogenic differentiation in the cells began by introducing an induction medium containing 7 fatty acids, F#9 (50 μM). Over a period of 7 days, the culture medium was changed every 3 days. After the differentiation period, one set of samples was fixed and stained with LipidTOX. The other set of samples was used for the quantification of lipids as described below (2.11).

### 4.11. Fluorometric Neutral Lipid Quantification Assay

Following the adipogenesis of BUSCs on GelMA-MPs and in GelMA-HG, samples underwent extraction with 750 μL of a chloroform (Fisher Chemical, HPLC Grade, Waltham, MA, USA)/methanol mixture (HPLC Grade) (2:1, *v*/*v*) under ultrasound in ice water for 30 min. After centrifuging at 4000× *g* at 4 °C for 5 min, supernatants were transferred to a new test tube with 187.5 μL of distilled water, vortexed, then centrifuged again at 4000× *g* at 4 °C for 5 min. Finally, 40 μL of the lower layer containing chloroform with extracted lipids was pipetted out for the lipid assay. For the standard curve, lipid concentrations ranging from 500 to 0 mg/dL were prepared through serial dilution using a standard solution of 20 g/dL (n = 9). The quantification of standards and samples was conducted in accordance with the manufacturer’s protocols (Cell Biolabs, San Diego, CA, USA). The solvent from both standards and samples was evaporated at 55 °C for 30 min. The extracted lipids were then cooled at 4 °C for 3 min, followed by the addition of 40 µL of isopropanol and 200 µL of fluorometric reagent. After vortexing, the samples were incubated in the dark at room temperature for 10 min. Subsequently, 100 μL of each sample was loaded onto 96-well plates, and the fluorescence of both samples and standards was measured at Ex/Em 490/525 nm using a TECAN Spark^®^ 10 M Plate reader, following the manufacturer’s instructions. The lipid contents of the samples (n = 4) were then calculated from the drawn standard curves.

### 4.12. DNA Quantification

Cells on GelMA-MPs or in GelMA-HG were digested with 0.5 mg/mL of collagenase in PBS for 2 h in the 37 °C incubator while shaking at 200 rpm. After digestion, samples were treated with a cell lysis buffer (Cell Signaling Technology, Danvers MA, USA) containing sodium dodecyl sulfate (SDS at 0.5%, Fisher Scientific, Hampton, NH, USA). The released DNA from BUSCs cultured on GelMA-MPs or in GelMA-HG were quantified using the Helixyte Green dsDNA Assay Kit (AAT Bioquest, Pleasanton, CA, USA) following the manufacturer’s protocol.

### 4.13. Statistical Analysis

For each experiment, at least 3 samples (n ≥ 3) were used as biological repeats, and data are presented as mean ± standard deviation. All experiments were performed independently at least twice. Only one representative experiment is shown. Statistical analysis was performed as described [58]. One-way ANOVA with a Tukey’s multiple comparisons test was performed to determine statistical significance using GraphPad Prism version 10.2.3 (347) (22 July 2024), GraphPad Software (La Jolla, CA, USA) for all quantitative data. Differences were considered significant at a *p*-value of <0.05.

## Figures and Tables

**Table 1:** Composition of nine different adipogenic differentiation media.

Formula	Medium	Inducers
#1	DMEM + 10% FBS	50 μM Indomethacin 0.5 μM Dexamethasone
#2	F1	20 μM Rosiglitazone
#3	F2	10 μg/mL Insulin
#4	DMEM + 10% FBS	0.5 mM IBMX 100 μM Indomethacin 1.0 μM Dexamethasone 20 μM Rosiglitazone
#5	Stem Pro	Commercial
#6	F1	10 μg/mL Insulin
#7 ^1^	F4	DMEM + 10% FBS + 10 μg/mL Insulin
#8	Stem Pro	0.5 mM IBMX 100 μM Indomethacin 1.0 μM Dexamethasone 20 μM Rosiglitazone 10 μg/mL Insulin
#9	DMEM + 10% FBS	50 μM Myristoleic Acid 50 μM Pristanic Acid 50 μM Phytanic Acid 50 μM Erucic Acid 50 μM Elaidic Acid 50 μM Oleic acid 50 μM Palmitoleic Acid

^1^ BUSCs were induced with F#4 for 7 days then cells were treated with the media consisting of DMEM + 10% FBS + 10 μg/mL insulin till the end of the adipogenesis.

**Table 2:** Compositions of inducers for pretreatment of BUSCs.

Ind.	Inducers
#1	0.5 mM IBMX + 0.5 μM Dexamethasone
#2	0.5 mM IBMX + 0.5 μM Dexamethasone + 50 μM Indomethacin
#3	0.5 mM IBMX + 0.5 μM Dexamethasone + 50 μM Indomethacin + 10 μg/mL Insulin
#4	0.5 mM IBMX + 1.0 μM Dexamethasone + 50 μM Indomethacin
#5	0.5 mM IBMX + 1.0 μM Dexamethasone + 10 μg/mL Insulin
#6	DMEM + 10%FBS

## Data Availability

All data and materials are available on request from the corresponding author. The data are not publicly available due to ongoing research using a part of the data.
